# ARID1A loss enhances sensitivity to c-MET inhibition by dual targeting of GPX4 and iron homeostasis, inducing ferroptosis

**DOI:** 10.1038/s41418-025-01510-x

**Published:** 2025-05-14

**Authors:** Xu Zhang, Zihuan Wang, Yilin He, Kejin Wang, Cheng Xiang, Yongfeng Liu, Yijiang Song, Aimin Li, Zhen Wang, Yingnan Yu, Wenxuan Peng, Side Liu, Joong Sup Shim, Changjie Wu

**Affiliations:** 1 Guangdong Provincial Key Laboratory of Gastroenterology, Department of Gastroenterology, Nanfang Hospital, Southern Medical University, Guangzhou, China; 2 Department of Radiation Oncology, Henan Provincial Key Laboratory of Radiation Medicine, The First Affiliated Hospital of Zhengzhou University, Zhengzhou, China; 3 Department of Laboratory Medicine, Guangdong Provincial Key Laboratory of Precision Medical Diagnostics, Guangdong Engineering and Technology Research Center for Rapid Diagnostic Biosensors, Guangdong Provincial Key Laboratory of Single-cell and Extracellular Vesicles, Nanfang Hospital, Southern Medical University, Guangzhou, China; 4 Cancer Centre, Faculty of Health Sciences, MOE Frontiers Science Centre for Precision Oncology, University of Macau, Taipa, Macau SAR China

**Keywords:** Tumour-suppressor proteins, Translational research, Drug development

## Abstract

ARID1A, a subunit of the SWI/SNF chromatin-remodeling complex, functions as a tumor suppressor in various cancer types. Owing to its high frequency of inactivating mutations, ARID1A has emerged as a promising target for the development of anticancer drugs. In this study, we report that ARID1A-deficient colorectal cancer (CRC) cells induce synthetic lethality when treated with inhibitors of c-MET receptor tyrosine kinase. c-MET specific inhibitor PHA-665752 as well as two other FDA-approved drugs, crizotinib and cabozantinib, selectively inhibited the growth of ARID1A-deficient CRC cells in vitro and in xenograft tumor models. Mechanistically, we identified a tripartite functional association among ARID1A, c-MET, and NRF2, where ARID1A and c-MET pathways converge on the NRF2 transcription factor, which regulates the transcription of GPX4, a key regulator of ferroptosis. ARID1A inactivation reduces c-MET expression, decreasing NRF2 nuclear localization and its binding to the GPX4 promoter, resulting in reduced GPX4 transcription. This creates a cellular dependency on the residual c-MET for minimal GPX4 expression to survive the ferroptotic cell death. Additionally, we demonstrate that ARID1A loss leads to increased intracellular labile iron accumulation by downregulating the iron-exporting protein SLC40A1, thereby increasing cellular susceptibility to ferroptosis. Inhibition of c-MET in ARID1A-deficient CRC cells diminishes GPX4 expression, resulting in elevated lipid peroxidation and glutathione depletion, ultimately inducing ferroptosis. This study reveals a novel synthetic lethal relationship between ARID1A and c-MET signaling in promoting ferroptosis and proposes c-MET inhibitors as a potential therapeutic strategy for ARID1A-deficient CRC.

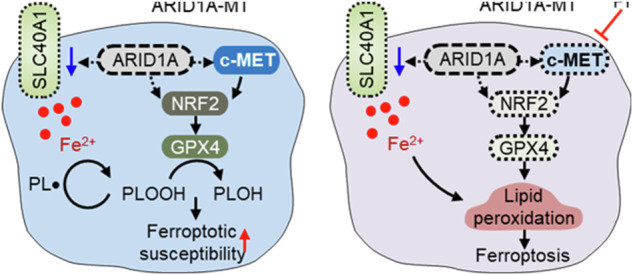

## Introduction

SWI/SNF complexes are evolutionarily conserved chromatin modulators that utilize energy from ATP hydrolysis to mobilize nucleosomes [1]. This complex exists in two major forms, BRG1-associated factor (BAF) and polybromo BAF [2]. Each complex is composed of ~15 protein subunits. With mutations in genes encoding SWI/SNF subunits, including *ARID1A*, *ARID1B*, *ARID2*, *PBRM1*, *SMARCA4*, and *SMARCB1*, collectively occurring in ~20% of all tumor types, SWI/SNF complexes are the most commonly mutated chromatin modulators in human cancer [3]. ARID1A (AT-rich interaction domain 1A), a subunit of the BAF complex, has been identified as one of the most frequently mutated tumor suppressors in a variety of cancers, with the highest mutation frequency in ovarian clear cell carcinoma (OCCC) [4] and endometrial cancer [5]. In colorectal cancer (CRC), the frequency of ARID1A mutations is ~10% [6]. However, mounting evidence has revealed that ARID1A loss also occurs at the epigenetic and transcriptional levels and is related to the clinicopathologic characteristics of CRC [7, 8]. According to immunohistochemistry analysis of patient sample data, ARID1A protein loss occurred in 25.8% of primary CRC tumors, and 51.2% of tumors had low ARID1A protein expression. The proportion of ARID1A protein loss was even higher as the tumor-node-metastasis stage advanced, up to 35.2% [7]. Furthermore, ARID1A loss is closely associated with DNA mismatch repair deficiency [8]. These data suggest that ARID1A is a key tumor suppressor in CRC and its loss is strongly linked to CRC progression and metastasis.

c-MET (mesenchymal-epithelial transition factor) is a type of receptor tyrosine kinase that is expressed on the surface of various epithelial cells [9]. Along with its ligand hepatocyte growth factor, c-MET can mediate embryogenesis, tissue regeneration, wound healing, and the formation of nerves and muscle [10–13]. However, as a type of proto-oncogene, abnormal activation of c-MET can promote the development and progression of multiple cancers such as liver [14], lung [15], colon [16], breast [17], pancreatic [18], ovarian [19], prostate [20], and gastric carcinomas [21] by stimulating the PI3K/AKT [22], Ras/MAPK [23], JAK/STAT [24], Wnt/β-catenin [25], and other signaling pathways. c-MET also plays an important role in cancer cell survival by promoting anti-apoptotic signals and protecting cells from oxidative stress by activating the NRF2 pathway [26]. Hence, c-MET is a well-recognized oncogenic drug target for anticancer drug development. Currently, a number of small-molecule drugs targeting c-MET are under clinical investigation, with two drugs, crizotinib and cabozantinib, receiving FDA approval. Crizotinib is approved by the FDA for the treatment of advanced non-small cell lung cancer and ROS1-positive lung cancer, whereas cabozantinib is approved for the treatment of progressive metastatic thyroid medullary carcinoma and advanced renal cell carcinoma.

Given the high frequency of ARID1A loss or inactivation in cancer, ARID1A loss has been increasingly exploited in cancer target discovery and precision oncology. Recent studies have shown that ARID1A has a synthetic lethal interaction with several cancer-associated proteins, including EZH2 [27], HDAC6 [28], GCLC [29], and AURKA [30]. Inhibition of these synthetic lethal targets resulted in selective vulnerabilities in ARID1A-mutant OCCC, CRC, and breast cancer cells. These studies demonstrate that synthetic lethal targeting of ARID1A is a promising approach for the development of novel cancer-targeted therapies. Based on this idea, we conducted a whole-kinase inhibitor library screen to identify synthetic lethal kinases for ARID1A loss in CRC cells. Here, we showed that c-MET inhibition induces selective vulnerability in ARID1A-deficient CRC cells in vitro and in vivo. We further explored the mechanism by which ARID1A loss and c-MET inhibition synergistically inactivate the expression of the antioxidant enzyme GPX4 by inhibiting NRF2 transcription factor, inducing lipid peroxidation and ferroptosis in ARID1A-deficient CRC cells. This study identified the c-MET-GPX4 axis as a potential drug target for CRC with ARID1A loss.

## Materials and methods

### Cell culture and reagents

CRC cell lines, HCT116, RKO, SW480, and LOVO, were obtained from the American Type Culture Collection (Manassas, VA) and authenticated by the manufacturer. HCT116 cells were cultured in the RPMI-1640 medium, while RKO, SW480, and LOVO cells were cultured in Dulbecco’s modified Eagle’s medium. RPMI-1640 medium and DMEM were supplemented with 10% fetal bovine serum (FBS) and 1% PS (penicillin/streptomycin). Cells were cultured in a humidified incubator maintained at 37 °C with 5% CO_2_. PHA-665752 (T6128), RSL3 (T3646), and erastin (T1765) were purchased from TargetMol (Waltham, MA, USA). Crizotinib (S1068) and cabozantinib (S1119) were purchased from Selleck Chemicals (Houston, TEXAS, USA).

### Kinase Inhibitor Library drug screening and cell viability measurement

The Kinase Inhibitor Compound Library (L1200) was purchased from Selleck Chemicals. Each compound was diluted by gradient and arrayed in 384-well plates in an 8-dose inter-plate titration format, ranging from 46 nM to 100 μM. Then, HCT116 *ARID1A*-WT or *ARID1A*-KO cells were seeded at 3000 cells per well in the 384-well plates containing working dilution of the compound library and incubated for 72 h at 37 °C CO_2_ incubator. When cell viability was measured, cells were incubated with 10% Alamar blue (Sigma-Aldrich, St. Louis, MO) in media solution for 2 h, and the fluorescence signal (ex560/em590) was read from the bottom of the plate and then recognized using SpectraMax-M4 (Molecular Devices, Sunnyvale, CA). The IC_50_ values of each compound against the ARID1A-isogenic cell pair were calculated using GraphPad Prism 8.0 (GraphPad Software, La Jolla, CA). We obtained the results of two independent experiments and counted the average IC_50_ value to identify synthetic lethality hits.

### siRNA silencing of *MET*

*MET* (si*MET* #1, si*MET* #2, and si*MET* #3) and *ARID1A* (si*ARID1A*) were designed and synthesized by RiboBio (Guangzhou, China). The sequences are as follows: si*MET* #1: 5′-GGACTTTGTTGGACAATGA-3′, si*MET* #2: 5′-CAATCATACTGCTGACATA-3′, si*MET* #3: 5′-GTCGGAGGTTCACTGCATA-3′, si*ARID1A* #1: 5′-GGACCTCTATCGCCTCTAT-3′. The transfection of siRNA was performed with Lipofectamine 3000, and the operation procedure was strictly following the manufacturer’s instructions. Briefly, after cells were trypsinized, 100 μM of siRNA was pipetted into 100 μL Opti medium and diluted to the indicated concentration, mixed with 100 μL Opti medium containing 1 μL Lipofectamine 3000, and the transfection mixture was incubated at room temperature for 20 min. A 1000 μL mixture of cells and medium containing serum was added to each well and mixed with the transfection mixture. Cells were then incubated for 72 h in a CO_2_ incubator, and cell images were taken using a U-HGLGPS (Olympus, Japan). Image J software was used to measure cell density to assess cell viability. The efficiency of siRNA knockdown was assessed by immunoblotting.

### Immunoblot and antibodies

Whole-cell protein was extracted with ice-cold RIPA buffer (25 mM Tris-HCl pH 7.6, 150 mM NaCl, 1% NP-40, 1% sodium deoxycholate, and 0.1% sodium dodecyl sulfate (SDS)). Electrophoresis of each protein sample was performed on an SDS-polyacrylamide gel and transferred onto a PVDF membrane (Millipore) for immunoblotting with primary antibodies, including ARID1A (Cell Signaling Technology, #12354S, 1:1000 dilution), NRF2 (Cell Signaling Technology, #12721S, 1:1000 dilution), c-MET (Cell Signaling, #8198S, 1:1000 dilution), GAPDH (FdBio, FD0063, 1:1000 dilution), GPX4 (Proteintech, 67763-1-Ig, 1:1000 dilution), SLC7A11 (Abcam, ab307601, 1:1000 dilution), FPN1 (Abcam, ab239583, 1:1000 dilution), and TFRC (Proteintech, 10084-2-AP, 1:1000 dilution) followed by horseradish peroxidase-conjugated secondary antibodies (ZEN-Bioscience, 511203). Uncropped versions of all blots are shown in Supplementary Figures.

### Tumor xenograft mouse model

All animal procedures were approved by the Animal Research Ethics Committee of the Southern Medical University (SMUL2021076) and all animals received humane acre according to the criteria outlined in the “Guide for the Care and Use of Laboratory Animals” (8th edition). Eight-week-old female BALB/c nude mice were implanted with *ARID1A*-WT (right flank) and *ARID1A*-KO (left flank) HCT116 cells carrying a mixture of Matrigel and phosphate-buffered solution (PBS). The mice were randomly divided into three groups (five mice per group), when both tumors were touchable, followed by treatment with equal tumor volume. Mice were treated with vehicle (sterile saline containing 5% dimethyl sulfoxide, 5% tween-80, and 5% polyethylene glycol-400) or PHA-665752 (15 and 30 mg kg^−1^ daily) via intravenous injection for 20 days. Tumor size was periodically measured with a Vernier caliper for 20 days, and tumor volume was calculated based on the modified ellipsoid formula (long axis × short axis × height axis × 0.53). At the end of the experiments, the mice were sacrificed, and the tumors were harvested for weighing and further analyses. The weights of the mice were measured regularly during the drug injection period to assess potential drug toxicity.

### RNA-sequencing analysis

Total RNA was extracted from HCT116 *ARID1A*-WT and *ARID1A*-KO cells using the RNeasy Kit (Qiagen, Germany, 74136). cDNA libraries were constructed using the NEBNext^®^ Ultra™ Directional RNA Library Prep Kit for Illumina^®^ (New England Biolabs, Ipswich, MA, USA, NEB #E7760). Following library preparation, the cDNA libraries were sequenced on an Illumina HiSeq platform (Illumina, San Diego, CA) at the Genomics and Bioinformatics Core Facility, Faculty of Health Sciences, University of Macau. The raw RNA-Seq data underwent initial quality control using FastQC to assess read quality. High-quality reads were retained for subsequent analysis. These quality-checked reads were aligned to the reference genome using TopHat 2.1.1 (Center for Computational Biology, Johns Hopkins University). Transcript abundance was quantified using Fragments Per Kilobase of transcript per Million mapped reads (FPKM) with Cufflinks 2.2.1 (Trapnell Lab, GitHub). Differential gene expression between *ARID1A*-WT and *ARID1A*-KO cells was determined, and functional annotation of the resulting transcriptome profiles was performed. Genes showing significant differential expression were subjected to KEGG (Kyoto Encyclopedia of Genes and Genomes) pathway analysis using the web-based tool DAVID v2023q4 (The Database for Annotation, Visualization, and Integrated Discovery, National Institute of Allergy and Infectious Diseases, NIH).

### ChIP of GPX4 promoter

Chromatin immunoprecipitation (ChIP) was performed using a Chromatin Immunoprecipitation Assay Kit (ChIP Assay Kit, P2078, Beyotime) according to the manufacturer’s instructions. Briefly, cells were treated with 1% formaldehyde, which was diluted from 270 μL 37% formaldehyde in 10 mL media for 10 min to crosslink DNA and proteins prior to preparing the nuclear fraction. To sonicate the nuclear pellet, SONICS VibraCell (Connecticut, US) was switched on and off for 30 s for 6 min to shear total DNA. The samples were then immunoprecipitated with ChIP-grade antibodies including anti-ARID1A (Cell Signaling Technology, #12354S, 2 μg per sample for ChIP), anti-NRF2 (Cell Signaling Technology, #12721S, 2 μg per sample for ChIP), anti-RNA polymerase II (Cell Signaling Technology, #2629S, 1 μg per sample for ChIP), and then the protein bound to protein was precipitated by protein A in the kit. Samples from the control groups were treated with ChIP-grade mouse IgG for immunoprecipitation. After reverse cross-linking and protein degradation of the samples, ChIP DNA was analyzed by qPCR against the GPX4 promoter region. Following are sequences of the primer pairs (integrated from Tsingke Biotechnology) used to analyze: ChIP DNA: Primer #1 (−1 to −250 bp from the TSS): forward primer 5′- AGCCGGATAACTGCGCTGCCTC-3′ and reverse primer 5′- GGACGCGCGTCGGCTTTCCGCG-3′; Primer #2 (−251 to −500 bp from the TSS):forward primer 5′-CTGTTGTCCCAGCTACTCGGGAAGC-3′ and reverse primer:5′-CCTGAGAATACTACTTAAGACTCG-3′; Primer #3 (−501 to −750 bp from the TSS): forward primer:5′-GTTGCAGTGAGCTGAGATAGC-3′ and reverse primer 5′-GCTCATCCACCATTCCTGGCTA-3′; Primer #4 (−751 to −1000 bp from the TSS): forward. primer 5′-CATTAAAAAGGCAAATCCCTTGGCC-3′ and reverse primer:5′-GTTCAAGCGATTCTCCTGACTCAGCCT-3′; and Control primer (ORF-free region): forward primer:5′-GGAGCGAGATCCCTCCAAAAT-3′ and reverse primer 5′-GGCTGTTGTCATACTTCTCATGG-3′. The ChIP-qPCR data were expressed as fold enrichment (ΔΔCt method), according to the manufacturer’s instructions. Briefly, the Ct value of each ChIP DNA fraction was normalized to the input DNA fraction Ct value (ΔCt [normalized ChIP]). ΔCt for antibody ChIP was normalized to the IgG control ChIP ΔCt (ΔΔCt [antibody ChIP/IgG ChIP]). Fold enrichment of a specific site was calculated according to the following equation: fold enrichment = 2^(−ΔΔCt [antibody ChIP/IgG ChIP])^.

### Assay of GSH and GSSG

The contents of GSH and GSSG in CRC cells were measured as described in the steps supplied with the GSH and GSSG Assay Kit (Beyotime, S0053, China). Briefly, after preparing reagents needed according to the instruction manual of the GSH and GSSG Assay Kit, cells were washed three times in precooled PBS, trypsinized, and vortexed to separate out cell precipitation. Cell precipitates which were dissolved in protein removal reagent M solution (Beyotime, S0053-5, China) were frozen in liquid nitrogen and thawed at 37 °C in a water bath. This procedure was repeated thrice. Then the samples were placed in an ice bath for 5  min and centrifuged at 10,000  rpm for 10  min at 4 °C. Then, the samples were separated into two parts, and one part of the samples was cleaned out of GSH to measure GSSG. The samples were added diluted GSH removal assist solution at a ratio of 20  µL per 100  µL samples and mixed immediately, the added GSH removal solution was at a ratio of 4  µL per 100  µL of samples, mixed immediately, and continued to react for 60  min at 25 °C. After the removal of GSH, according to the 5, 59 dithiobis (2-nitrobenzoic acid)-glutathione disulfide (DTNB-GSSG) reductase-recycling assay,150  μL of detection solution was added to 96-well plates, and then 10  μL of samples was pipetted into each well. After the dishes were equilibrated at 25 °C for 5 min, a total of 50 μL 0.16 mg/mL NADPH was added to each well. Because 5-thio-2-nitrobenzonic acid has an absorption peak at 412 nm, after 5 min reaction samples were monitored with a spectrophotometer at a fluorescence signal of 412 nm at the beginning and after 25 min. Standards (0.5, 1, 2, and 10 mM) of glutathione and a sample blank lacking glutathione were assayed simultaneously. The GSH content was calculated according to the following formula: GSH = Total Glutathione−GSSG × 2.

### Assay of intracellular ROS levels

The reactive oxygen species (ROS) levels in CRC cells were measured using a Reactive Oxygen Species Assay Kit (Beyotime Biotechnology, S0033S, China); the key component of which is 2′,7′-dichlorofluorescein-diacetate (DCFH-DA), which can be easily oxidized to fluorescent dichlorofluorescein (DCF) by intracellular ROS to measure ROS. Briefly, after cells were seeded for 24 h, they were treated with DMSO or 6 μM PHA for 48 h. DCFH-DA was diluted to 10 μM with PBS. The cells were incubated with a detection solution of 1 mL per well avoiding light for 20 min at 37 °C, and then observed by fluorescence microscopy U-HGLGPS (Olympus, Japan).

### Measurement of intracellular iron levels

Intracellular iron levels were measured using calcein AM (MCE, HY-D0041, US). Briefly, after seeded for 24 h, cells were treated with DMSO or 6 μM PHA for 48 h. Following the treatment, the cells were incubated with calcein, which was diluted to 10 μM in PBS, and avoided light for 20 min at 37 °C. Calcein-AM fluorescence was observed using a Beckman Coulter flow cytometer (Beckman Coulter, California, US) with a FITC detector, and data were analyzed using Flow-Jo (BD, California, USA).

### Assay of BODIPY 581/591 C11

BODIPY 581/591 C11 (Thermo Fisher, D3861, USA) was used to detect ROS in cells and cell membranes. Briefly, after seeded for 24 h, cells were treated with DMSO or 6 μM PHA for 48 h. Following the treatment, the cells were incubated with BODIPY 581/591 C11 for 1 h at 37 °C, which was diluted to 10 μM. Fluorescence was observed using a Beckman Coulter flow cytometer (Beckman Coulter, California, US) with a FITC detector.

### RT and qPCR

Total cellular RNA was extracted from cracked cells using TRIzol (Thermo Fisher Scientific) to crack cells. RT was performed with the High-Capacity cDNA Reverse Transcription Kit (Thermo Fisher Scientific). Gene transcription levels were determined with SYBR Green Supermix (Bio-Rad, Hercules, CA) with the primer synthesized by Tsingke Biotechnology (Peking, China). Sequence of primers was listed in Supplementary Table 1.

### Immunoprecipitation of NRF2

HCT116 cells were cultured in RPMI medium supplemented with 10% FBS. The cells were washed three times with PBS, lysed in RIPA buffer, collected, and used for immunoprecipitation with 4 μg anti-NRF2 (Cell Signaling Technology, #12721S) specific for the IP group and 4 μg anti-IgG (Proteintech, 30000-0-AP, China) specific for the IgG group. The precipitated and input samples were analyzed by immunoblot analysis.

### Immunofluorescence analyses

Cells were seeded in a 15 mm confocal cell dish (Nest, China) and treated with 4 μM PHA or DMSO for 72 h. The cells were then fixed with 4% paraformaldehyde, permeabilized with 0.25% Triton X-100, washed three times with PBS, and blocked with 5% bovine serum albumin for 1 h. Cells were incubated with anti-NRF2 (Cell Signaling Technology, #12721S, 1:1000 dilution) in the blocking buffer overnight at 4 °C, followed by the incubation with secondary antibody (Beyotime, A0413, 1:1000 dilution) conjugated with Alexa Fluor 488 for 1 h at room temperature. Nuclei were stained with DAPI (Beyotime, P0131). The cells were observed under an Olympus FluoView™ Fv1000 microscope (Olympus, Japan).

### Statistical analysis

All data are presented as mean ± standard deviation (s.d.). Data analysis was performed using GraphPad Prism 8 software (GraphPad Software, La Jolla, CA, USA). The differences between the control and test groups were analyzed using Student’s *t*-test and one-way analysis of variance (ANOVA); statistical significance was set at *P* < 0.05. ANOVA using GraphPad Prism 8 showed different statistical analyses between the two dose-response curves.

## Results

### Kinase inhibitor library screen identifies c-MET inhibitors as synthetic lethal drugs for ARID1A loss

**Fig. 1:**
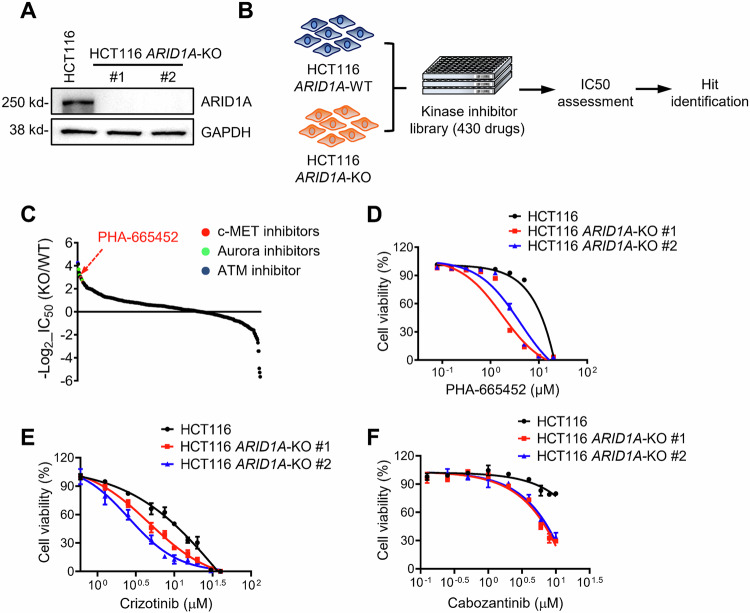
Screening of Kinase inhibitor library for synthetic lethality in HCT116 cell. **A** Immunoblot analysis showing loss of ARID1A expression in the two *ARID1A*-KO clones. **B** Schematic illustration of the synthetic lethal kinase inhibitor screening. HCT116 *ARID1A*-WT and *ARID1A*-KO cell lines were screened in parallel with 430 kinase inhibitor library in an 8-dose titration format. After incubation with the compound library for 72 h, cell viability was determined by Alamar Blue assay. **C** A −log2 IC50 plot of the screening results. −log2 scale of IC50 values of the drugs against HCT116 *ARID1A*-KO and *ARID1A*-WT cells was plotted. **D** Dose-response curves of HCT116 *ARID1A*-WT and two *ARID1A*-KO clones treated with c-MET kinase inhibitor for 72 h are shown. Error bars represent s.d. (*n* = 3) from three independent experiments. Dose-response curves of HCT116 *ARID1A*-isogenic cell pair treated with c-MET kinase inhibitors crizotinib (**E**) and cabozatinib (**F**) are shown. HCT116 *ARID1A*-WT, *ARID1A*-KO #1, and *ARID1A*-KO #2 clones were incubated with indicated compounds for 72 h and the cell viability was determined by Alamar Blue assay. Error bars represent s.d. (*n* = 3) from three independent experiments.

To identify ARID1A synthetic lethal targets, we used the ARID1A-isogenic HCT116 CRC pair that we previously generated using the CRISPR-Cas9 system [30]. The ARID1A status in the isogenic pair was verified by immunoblot analysis (Fig. 1A). Although identified as a tumor suppressor, ARID1A loss does not enhance cell proliferation in short-term cell culture, as several papers have reported [27, 28, 31]. We then conducted a chemical screening of a kinase inhibitor library containing 430 small-molecule inhibitors targeting various human kinases against the ARID1A-isogenic HCT116 pair. The screening was performed in an 8-dose titration format, with a working concentration range from 0.009 to 20 μM to obtain IC50 values of drugs for each cell line (Fig. 1B). ATM inhibitor (KU-60019), aurora kinase A (AURKA) inhibitors (AMG-900, VX-680, MK-5108), and c-MET inhibitors (PHA-665752, MK-2461) were identified as the top synthetic lethal drugs among the kinase inhibitors screened (Fig. 1C; Supplementary Fig. 1). As ATM [32] and AURKA [30] have been reported to have a synthetic lethal interaction with ARID1A in CRC, we selected c-MET as a novel synthetic lethal target for ARID1A in CRC for follow-up studies. We validated the synthetic lethal effect of the c-MET inhibitor PHA-665752 (PHA) in two *ARID1A* knockout (*ARID1A*-KO) clones, where PHA selectively inhibited the growth of *ARID1A*-KO HCT116 cells (Fig. 1D). Moreover, two FDA-approved c-MET inhibitors, crizotinib, and cabozantinib, also showed synthetic lethality in ARID1A-KO HCT116 clones (Fig. 1E, F). These results suggest that c-MET is a potential synthetic lethal target for ARID1A in CRC cells.

**Fig. 2:**
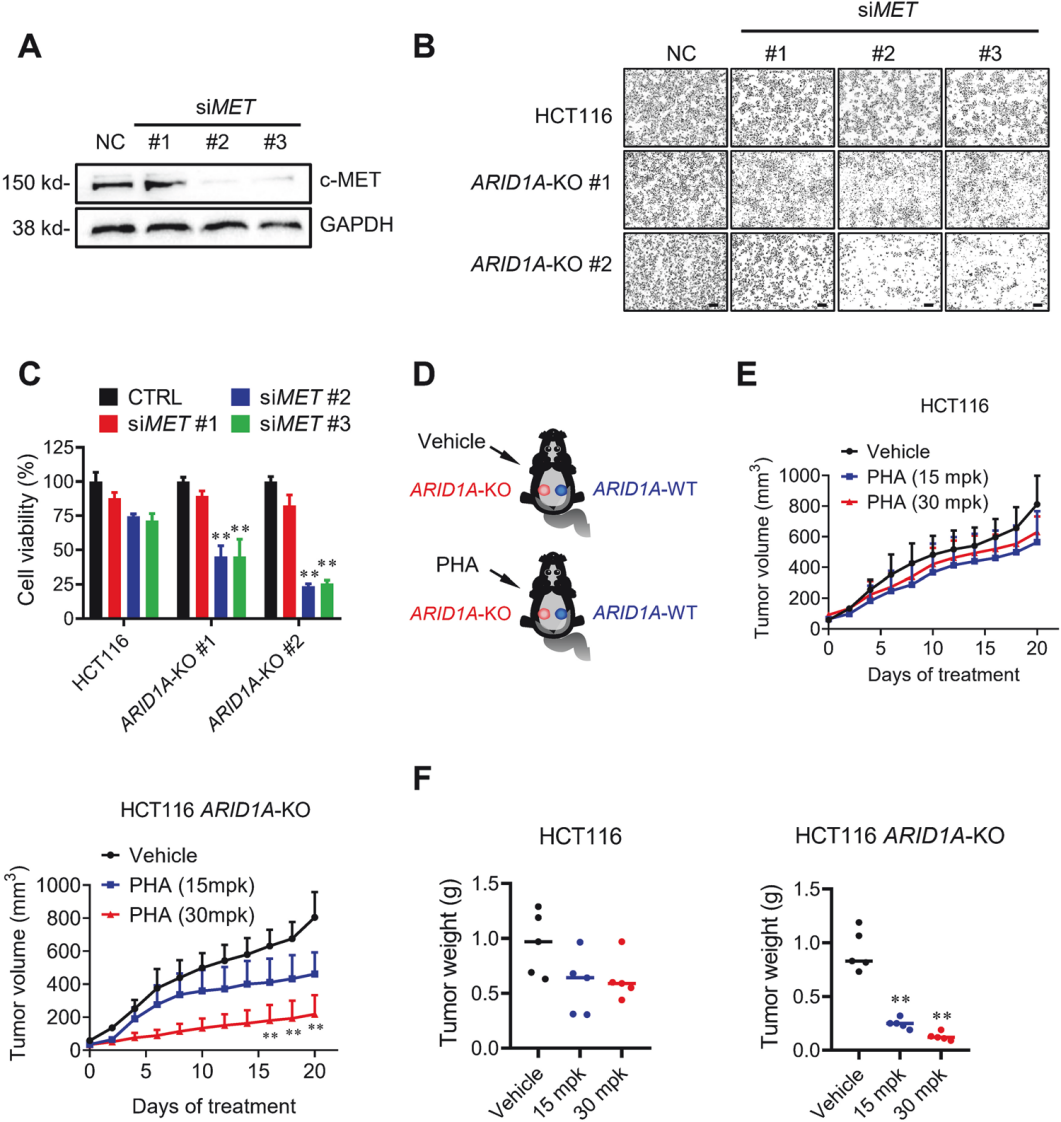
In vitro and in vivo synthetic lethality in *ARID1A*-KO HCT116 cells by c-MET kinase inhibitor. **A** Silencing of *MET* expression in HCT116 by siRNA (si*MET*). GAPDH was used as a control. **B** Synthetic lethality validation with si*MET*. *ARID1A*-WT and *ARID1A*-KO clone was transfected with 100 μM si*MET* for 72 h and the cell images were taken. Scale bars, 100 μm. **C** The cell density was determined with Image J software. ANOVA *P* value of <0.01. **D** Schematic illustration of mouse tumor xenograft experiments with HCT116 ARID1A-isogenic cell pair. **E** Tumor growth curve in nude mice bearing HCT116-WT or HCT116 *ARID1A*-KO xenografts after intravenous injection of vehicle, 15 or 30 mg kg^−1^ (mpk) PHA. Error bars represent a *P* value of <0.01 between vehicle and PHA treatment groups (*n* = 3). Student’s *t*-test. **F** Wet weight measurement of the tumors isolated from mice bearing HCT116-WT or HCT116 *ARID1A*-KO xenografts at 24 days after injection of vehicle, 15 or 30 mpk PHA. Error bars represent s.d.

To validate c-MET as a synthetic lethal target of ARID1A in CRC cells, we silenced c-MET expression using a specific siRNA and analyzed its synthetic lethal effect in the ARID1A-isogenic CRC pair. Similar to the results of c-MET inhibitor treatment, depletion of c-MET expression by siRNA selectively inhibited the growth of *ARID1A*-KO HCT116 cells (Fig. 2A–C). These data demonstrate that c-MET kinase inhibition is likely attributable to synthetic lethality with ARID1A loss in CRC cells. Next, we investigated the synthetic lethal effect of c-MET inhibitors in vivo using a tumor xenograft mouse model. Athymic nude mice bearing ARID1A-isogenic HCT116 tumors on either flank were intravenously administered PHA at dosages of 15 and 30 mg kg^−1^ for 20 consecutive days. Tumor volume was periodically measured until day 20 after the first drug administration (Fig. 2D). PHA showed a weak antitumor effect on ARID1A wild-type tumors, while it significantly inhibited the growth of ARID1A-KO tumors under the same dosage regimen (Fig. 2E, F, Supplementary Fig. 2). PHA did not appear to cause toxicity in mice under the same treatment conditions, as assessed by changes in body weight (Supplementary Fig. 2). These results demonstrated that ARID1A-deficient CRC tumors are highly vulnerable to c-MET kinase inhibitor treatment in vitro and in vivo.

### ARID1A-deficient CRC cells are susceptible to ferroptosis induction

**Fig. 3:**
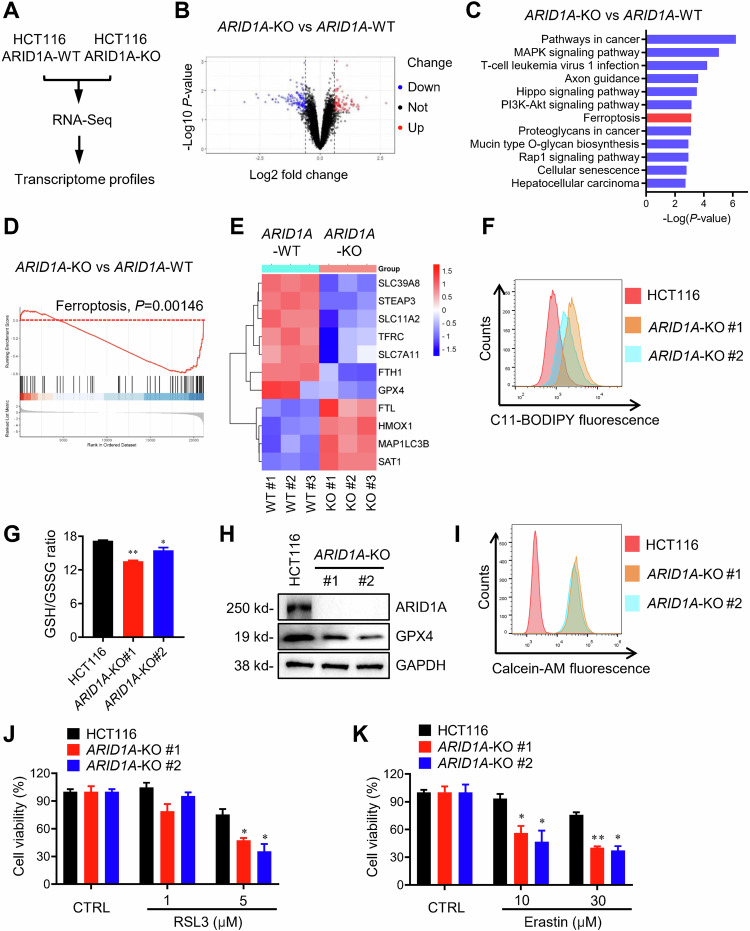
Knocking out ARID1A activates the ferroptosis signaling pathway. **A** Schematic illustration of RNA-seq contrasting with HCT116-WT and HCT116 *ARID1A*-KO cells. **B** volcano plot depicting the results of the RNA-Seq study. **C** Top 12 enrichment scores of KEGG pathway enrichment analysis. **D** GSEA analysis on *ARID1A*-KO vs. *ARID*1A-WT samples of the ferroptosis gene set. **E** The heat map data showed gene expression alteration of ferroptosis-correlated molecules after *ARID1A* knock out including GPX4, SLC7A11, FTH1, HMOX1, and so on. **F** Lipid peroxidation level was detected in HCT116-WT and two HCT116 *ARID1A*-KO clones with C11-BODIPY (581/591). **G** The ratio of GSH with GSSG in HCT116 and HCT116 *ARID1A*-KO cells was detected. **H** GPX4 expression level was detected with western blot analysis in HCT116-WT and two HCT116 *ARID1A*-KO clones. **I** Cellular labile iron pool (LIP) was detected by calcein-AM reporter in in HCT116-WT and two HCT116 *ARID1A*-KO clones. HCT116-WT and two HCT116 *ARID1A*-KO clones were treated with ferroptosis inducers RSL3 (**J**) and Erastin (**K**) for 72 h. The cell viability was determined by Alamar Blue assay. Error bars represent s.d. ANOVA *P* value < 0.01.

ARID1A is a component of the chromatin remodeler that participates in a variety of cellular processes by regulating gene transcription. However, there is no clear functional connection with the c-MET receptor tyrosine kinase pathway in CRC cells. To investigate the functional crosstalk between ARID1A and c-MET, we conducted RNA-Seq transcriptome profiling of *ARID1A*-WT and *ARID1A*-KO HCT116 cells and analyzed the major pathways affected by ARID1A status. As a result, a total of 716 differentially expressed genes (DEGs) were obtained from the RNA-Seq dataset (Fig. 3A, B). KEGG pathway analysis revealed that ferroptosis was one of the pathways most affected by ARID1A status (Fig. 3C). Gene set enrichment analysis (GSEA) of the DEGs also showed that the transcripts involved in ferroptosis were particularly modulated in *ARID1A*-KO cells (Fig. 3D). The constructed heat map showed that ARID1A loss led to significant changes in 11 genes closely related to ferroptosis, including downregulation of anti-ferroptotic genes GPX4 and FTH1, and upregulation of pro-ferroptotic genes HMOX1 and SAT1 (Fig. 3E). These data suggested that ARID1A loss might promote cellular ferroptotic environment. Ferroptosis has been described as iron-dependent and ROS-dependent cell death. Ferroptotic cell death results from the loss of selective membrane permeability of the plasma membrane due to membrane lipid peroxidation and the imbalance of redox homeostasis. Given that c-MET plays an important role in protecting cells from oxidative damage by regulating the cellular redox system [26], we hypothesized that c-MET inhibition might sensitize ARID1A-deficient cells to ferroptosis. To test this hypothesis, we first investigated the role of ARID1A in ferroptosis using ferroptosis markers, including lipid peroxidation, glutathione (GSH) depletion, and glutathione peroxidase 4 (GPX4) downregulation [33]. The level of cellular lipid peroxidation was measured using the C11-BODIPY (581/591) fluorescent reporter, which shifts its fluorescence when the polyunsaturated butadienyl portion of the dye is oxidized by ROS. The results of the fluorescent reporter assay showed that *ARID1A*-KO cells had a significantly increased lipid peroxidation rate (Fig. 3F). The level of GSH (reduced form of glutathione) was detected by measuring the ratio of GSH to oxidized glutathione disulfide (GSSG) using the chromogenic substrate DTNB. *ARID1A*-KO cells had a significantly lower GSH/GSSG ratio than *ARID1A*-WT CRC cells (Fig. 3G). Notably, GPX4 expression levels were downregulated in *ARID1A*-KO cells compared to *ARID1A*-WT CRC cells (Fig. 3H). Furthermore, an increased cellular labile iron pool (LIP), which was detected by calcein-AM reporter, was also observed in *ARID1A*-KO cells (Fig. 3I). These results suggested that ARID1A-deficient cells are highly susceptible to ferroptosis. Indeed, *ARID1A*-KO HCT116 cells were significantly more sensitive to ferroptosis-inducing compounds, including RSL3 and erastin, than *ARID1A*-WT cells (Fig. 3J, K).

On the other hand, the transferrin receptor (TFRC), a key regulator in cellular iron uptake, showed a decrease in its expression in *ARID1A*-KO cells (Fig. 3E; Supplementary Fig. 3A, B). This is unexpected because the decrease in TFRC would typically result in lower labile iron levels [34]. To address this discrepancy, we analyzed the effect of ARID1A on the levels of iron-exporting proteins. Given that SLC40A1 (also known as ferroportin) is the only known transporter responsible for exporting intracellular iron in mammals [35], we examined SLC40A1 expression in ARID1A wild-type and KO cells. RT-qPCR and Western blot data showed a dramatic reduction in SLC40A1 expression in *ARID1A*-KO cells (Supplementary Fig. 3A, B), suggesting that the increased intracellular labile iron observed in ARID1A-deficient cells results from a reduction in iron export. We believe that the reduction in TFRC levels in *ARID1A*-KO cells is presumably a cellular defense mechanism for survival under high levels of intracellular labile iron.

### The synthetic lethality of ARID1A and c-MET is mediated by ferroptosis

**Fig. 4:**
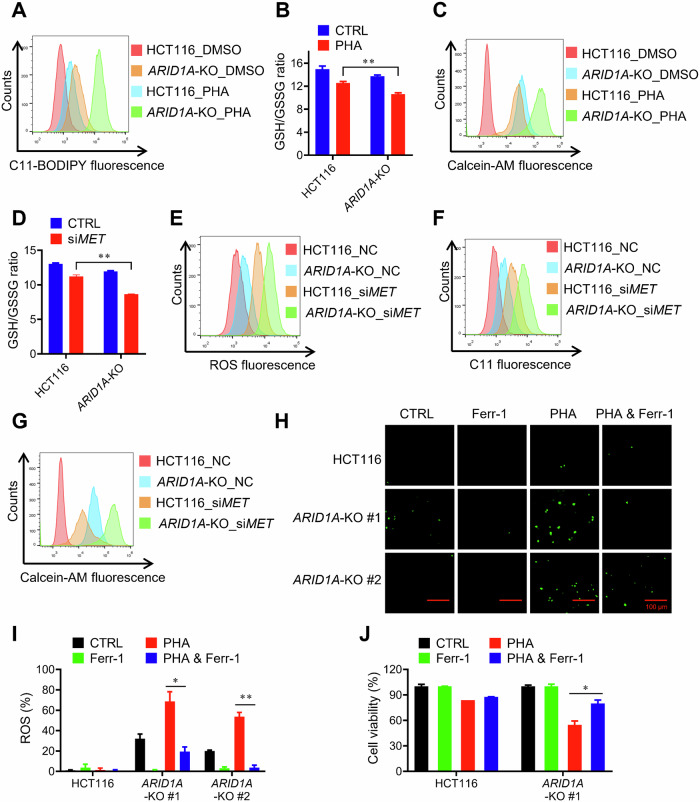
Synthetic lethality is ferroptosis pathway dependent. **A** HCT116-WT and HCT116 *ARID1A*-KO cells were treated with or without PHA for 72 h. Lipid peroxidation level was conducted with C11-BODIPY assay. **B** c-MET inhibition reduced the ratio of GSH/GSSG in HCT116 and HCT116 *ARID1A*-KO cells. **C** PHA treatment raised iron accumulation, especially in *ARID1A*-KO cells. **D** Transfection with 100 μM si*MET* for 48 h reduced the ratio of GSH with GSSG in HCT116 and HCT116 *ARID1A*-KO cells. Transfection with 100 μM si*MET* for 48 h raised ROS intensity (**E**) lipid peroxidation (**F**) and iron accumulation (**G**) in HCT116 and HCT116 *ARID1A*-KO cells. **H**, **I** HCT116 and HCT116 *ARID1A*-KO cells were treated with or without 4 μM PHA, and 20 μM ferrostatin-1 (Ferr-1) for 72 h, and ROS intensity was analyzed with fluorescence. The fluorescence intensity was calculated with Image J software. Scale bars, 100 μm. **J** HCT116 and HCT116 *ARID1A*-KO cells were treated with or without 4 μM PHA, 20 μM Ferr-1 for 72 h. The cell viability was determined by Alamar Blue assay. Error bars represent s.d. from three independent experiments. ANOVA *P* value of <0.001.

Based on our observations, we postulated that ARID1A deficiency increases cellular susceptibility to ferroptosis, and c-MET plays a protective role against ferroptosis induction in ARID1A-deficient CRC cells. To test this hypothesis, we first tested the effects of the c-MET inhibitors PHA and c-MET siRNA on ferroptosis induction in ARID1A-isogenic CRC. Our results showed that lipid peroxidation levels were higher in *ARID1A*-KO cells than in *ARID1A*-WT cells, and PHA treatment significantly increased lipid peroxidation in *ARID1A*-KO cells (Fig. 4A). GSH depletion and the increase in LIP levels were also significantly promoted by PHA treatment in *ARID1A*-KO cells (Fig. 4B, C). Similar results were obtained with c-MET siRNA, where c-MET depletion significantly promoted ROS generation, lipid peroxidation, GSH depletion, and LIP levels in *ARID1A*-KO CRC cells (Fig. 4D–G). We then tested the effect of the specific ferroptosis inhibitor ferrostatin-1 on the synthetic lethal effect of the c-MET inhibitor in ARID1A-deficient CRC cells. Similar to the effect of c-MET siRNA, PHA significantly increased ROS levels in *ARID1A*-KO cells (Fig. 4H, I). This effect was significantly reversed by ferrostatin-1 co-treatment. Furthermore, ferrostatin-1, as well as the iron chelator deferoxamine (DFO) significantly rescued the PHA-induced cell death in *ARID1A*-KO cells (Fig. 4J; Supplementary Fig. 3C, D). Ferrostatin-1 also successfully rescued the c-MET siRNA-induced synthetic lethality in *ARID1A*-KO cells (Supplementary Fig. 4). These data strongly support the idea that ARID1A-deficient cells are highly susceptible to ferroptosis, and c-MET inhibition sensitizes ARID1A-deficient cells to ferroptotic cell death.

**Fig. 5:**
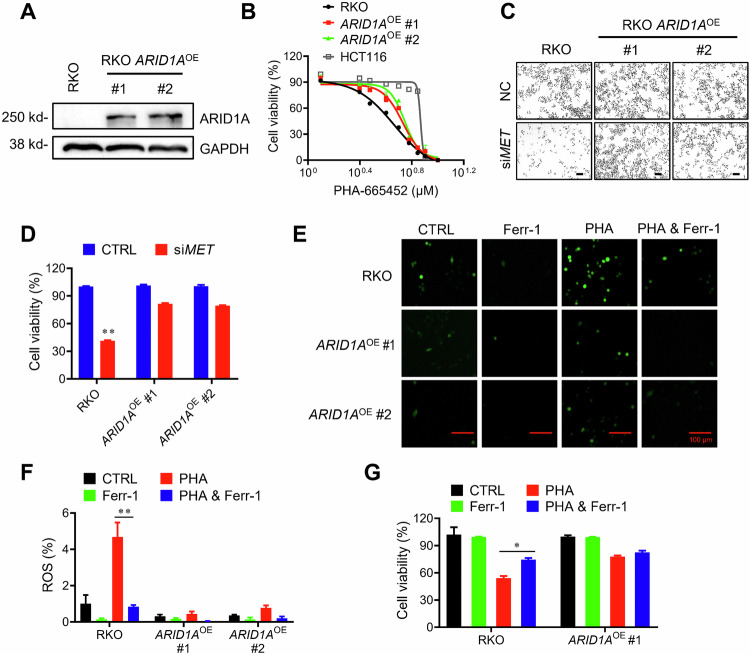
Synthetic lethality and ferroptosis in ARID1A-deficient RKO cells by PHA treatment. **A** Expression level of ARID1A in RKO and two selected ARID1A stable clones were detected with immunoblot analysis. **B** Dose-response curves of parental RKO and ARID1A-overexpressing (*ARID1A*^OE^) RKO clones treated with PHA. HCT116 *ARID1A*-WT cells were used as a positive control. Error bars represent s.d. (*n* = 5) from three independent experiments. **C** Synthetic lethality with si*MET* transfection in RKO isogenic cells. RKO and *ARID1A*^OE^ clones were transfected with 100 μM si*MET* for 72 h. Scale bars, 100 μm. **D** The cell density was determined with Image J software. ANOVA *P* value of <0.01. **E**, **F** RKO and *ARID1A*^OE^ clones were treated with or without PHA and Ferr-1. ROS intensity was analyzed with fluorescence. The fluorescence intensity was calculated with Image J software. Scale bars, 100 μm. **G** RKO and *ARID1A*^OE^ clones were treated with or without PHA and Ferr-1. The cell viability was determined by Alamar Blue assay. Error bars represent s.d. from three independent experiments. ANOVA *P* value of <0.01.

To further validate the ferroptosis-mediated synthetic lethality of ARID1A and c-MET, we established another ARID1A-isogenic cell model derived from the RKO CRC cell line, which has a frameshift mutation in ARID1A [30]. We reintroduced wild-type *ARID1A* into RKO cells with lentiviral transduction to establish an ARID1A-isogenic cell pair (Fig. 5A). Consistent with the HCT116 ARID1A-isogenic cell pair data, PHA and c-MET siRNA showed synthetic lethal effects in ARID1A-mutant RKO cells (Fig. 5B–D). Moreover, PHA significantly increased ROS levels in ARID1A-mutant RKO cells, and this effect was largely diminished by ferrostatin-1 treatment (Fig. 5E, F). Similarly, PHA-induced cell death in ARID1A-mutant RKO cells was significantly rescued by ferrostatin-1 co-treatment (Fig. 5G), further supporting our hypothesis that the synthetic lethality of ARID1A and c-MET is largely mediated by ferroptosis.

### ARID1A transactivates the expression of GPX4, a master regulator of ferroptosis

**Fig. 6:**
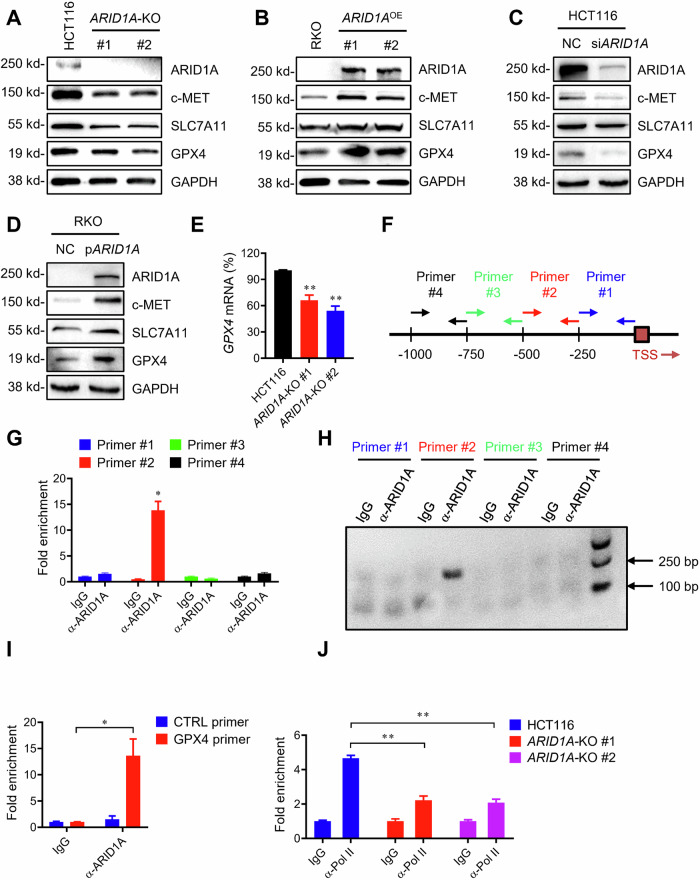
GPX4 is a target gene for transcription repression by ARID1A. **A** Downregulation of c-MET, SLC7A11, and GPX4 level in *ARID1A*-KO HCT116 cells. **B** Upregulation of c-MET, SLC7A11, and GPX4 level in *ARID1A*-overexpressing *(ARID1A*^OE^) RKO clones. **C** Silencing ARID1A expression decreases c-MET, SLC7A11, and GPX4 levels in HCT116 cells. **D** Upregulation of c-MET, SLC7A11, and GPX4 level in RKO cells by ARID1A overexpression plasmid transfection. **E** RT-qPCR analysis of *GPX4* mRNA level in HCT116 and *ARID1A*-KO clones. ANOVA *P* < 0.01. **F** Four primers (Primer #1, Primer #2, Primer #3, Primer #4) were designed to cover GPX4 promoter from −1 bp to −1000 bp. **G** Chip of GPX4 promoter in HCT116 cells using anti-ARID1A antibody. **H** A representative image showing results of PCR performed on DNA samples precipitated with anti-ARID1A. **I** ChIP of GPX4 promoter in HCT116 cells using anti-ARID1A antibody. IgG was used as a normalization control. ANOVA *P* < 0.05. **J** ChIP of GPX4 promoter in HCT116 and *ARID1A*-KO cells using anti-Pol II antibody. IgG in each cell line was used as a normalization control. ANOVA *P* < 0.01.

To study the in-depth mechanism of synthetic lethality, we investigated the interplay between ARID1A and c-MET and their roles in ferroptosis induction in CRC cells. Previous studies have shown that ARID1A, as a component of the SWI/SNF complex, could transactivate c-MET and SLC7A11 and play a role in the cellular antioxidant mechanism [29, 36]. SLC7A11 is an antiporter that mediates the uptake of extracellular cysteine in exchange for glutamate, thereby maintaining the balance between GSH and ROS. Consistent with these reports, we observed that basal levels of c-MET and SLC7A11 were lower in ARID1A-deficient cells than in their wild-type counterparts (Fig. 6A). More importantly, we noticed that GPX4 levels were downregulated in ARID1A-deficient cells (Figs. 3H and 6A). GPX4 is an antioxidant enzyme that uses GSH to reduce lipid peroxidation and thus acts as a master regulator of ferroptosis [37]. GPX4 inhibition is known to increase cellular ferroptosis sensitivity [38, 39]. Therefore, we investigated the role of ARID1A in GPX4 expression in greater detail. Stable overexpression of wild-type ARID1A in ARID1A-deficient RKO cells resulted in an increase in GPX4 expression, along with other potential ARID1A target genes, such as c-MET and SLC7A11 (Fig. 6B). To rule out the possibility that the GPX4 downregulation by ARID1A was due to a secondary effect during cellular adaptation follow long-term ARID1A deficiency, we tested the effect of transient depletion or overexpression of ARID1A on GPX4 expression. Transient depletion of ARID1A using siRNA effectively downregulated GPX4 level in ARID1A-wild-type cells (Fig. 6C). Furthermore, transient overexpression of wild-type ARID1A in ARID1A-deficient RKO cells increased GPX4 expression levels (Fig. 6D). The expression of c-MET and SLC7A11 was similarly regulated upon ARID1A depletion or overexpression (Fig. 6C, D; Supplementary Fig. 5). These results imply that GPX4 may be a direct downstream transcriptional target of ARID1A. To determine whether GPX4 expression is transcriptionally regulated by ARID1A, we first conducted an RT-qPCR analysis of GPX4 mRNA in ARID1A-isogenic cell pairs. RT-qPCR analysis showed that two HCT116 *ARID1A*-KO clones exhibited a significant decrease in GPX4 mRNA levels compared to their *ARID1A*-WT counterparts (Fig. 6E), demonstrating that GPX4 expression is transcriptionally regulated by ARID1A. To investigate whether GPX4 is a direct transcriptional target of ARID1A, we conducted ChIP experiments using a ChIP-grade ARID1A antibody and primer pairs targeting the GPX4 promoter region. We designed four pairs of primers that largely covered the entire GPX4 promoter region of the genome (Fig. 6F). Among the primers used, primer #2 target region, which covers the region from −251 to −500 bp upstream of the GPX4 transcription start site, was significantly enriched by ARID1A-ChIP (Fig. 6G–I). These results suggest that ARID1A binds directly to the GPX4 promoter region. To investigate whether the binding of ARID1A to the GPX4 promoter could positively regulate the transcription of GPX4, we conducted RNA-Pol II ChIP on the GPX4 promoter. Indeed, RNA-Pol II binding to the GPX4 promoter was reduced in *ARID1A*-KO cells compared to wild-type cells (Fig. 6J), indicating that ARID1A binds to the GPX4 promoter and transactivates its expression.

### GPX4 is a common target of ARID1A and c-MET signaling in ferroptosis induction and synthetic lethality

**Fig. 7:**
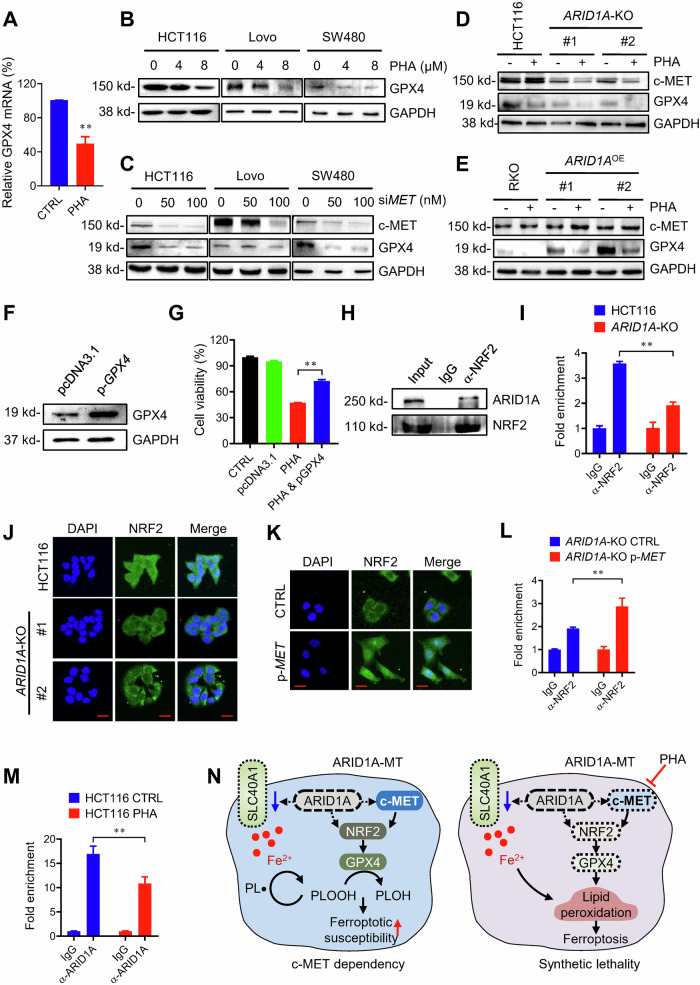
c-MET/NRF2/GPX4 axis is a target for synthetic lethality in *ARID1A*-KO colorectal cancer cells. **A** RT-qPCR analysis of *GPX4* mRNA level with PHA treatment in HCT116 cells. ANOVA *P* < 0.01. **B** Immunoblot analysis showing PHA treatment downregulates GPX4 level in HCT116, Lovo, and SW480 cells. **C** c-MET silencing induces GPX4 downregulation in HCT116, Lovo, and SW480 cells. Inhibition of GPX4 level by PHA treatment in HCT116 (**D**) and RKO (**E**) cells. **F**, **G** Overexpression of GPX4 reversed synthetic lethality induced by PHA treatment in HCT116 *ARID1A*-KO cells. HCT116 *ARID1A*-KO cells were treated with or without PHA and GPX4 expression plasmid. The cell viability was determined by Alamar Blue assay. ANOVA *P* value of <0.01. **H** co-IP showing ARID1A binds with NRF2 in HCT116 cells. **I** ChIP of GPX4 promoter in HCT116 and *ARID1A*-KO cells using anti-NRF2 antibody. IgG in each cell line was used as a normalization control. ANOVA *P* < 0.01. **J** Immunofluorescences showing that ARID1A loss attenuates NRF2 nuclear localization in HCT116 cells. Scale bars, 20 μm. **K** c-MET overexpression promotes NRF2 nuclear localization in *ARID1A*-KO cells. Immunofluorescences show that c-MET overexpression promotes NRF2 nuclear localization in *ARID1A-KO* cells. Scale bars, 20 μm. **L** c-MET overexpression promotes the binding between NRF2 and GPX4 promoter. ChIP of GPX4 promoter in *ARID1A*-KO cells using anti-NRF2 antibody with or without c-MET expression plasmid. IgG in each cell line was used as a normalization control. ANOVA *P* < 0.01. **M** ChIP of GPX4 promoter in HCT116 cells using anti-ARID1A antibody with or without PHA treatment. IgG in each cell line was used as a normalization control. ANOVA *P* < 0.01. **N** Working model of the synthetic lethality. In *ARID1A*-WT cells, ARID1A promotes c-MET transcription and cooperatively regulates NRF2 transcription factor functions with c-MET, thereby promoting GPX4 transcription. ARID1A inactivation downregulates c-MET and GPX4 levels, creating a cellular dependency on the residual activity of c-MET and GPX4. ARID1A inactivation also downregulates iron-exporting protein SLC40A1, increasing intracellular iron accumulation and lipid peroxidation. Pharmacological inhibition of c-MET (such as PHA665452) in ARID1A-deficient CRC cells diminishes GPX4 transcription, resulting in increased lipid peroxidation and ultimately inducing ferroptotic cell death. PLOOH a reactive phospholipid hydroperoxide, PLOH non-reactive alcohol, PL• a carbon-centered radical on a phospholipid chain.

We showed that GPX4 level was downregulated in ARID1A-deficient CRC cells, which potentially promoted ferroptosis sensitivity in this cell type. c-MET is also known to play a key role in antioxidant defense mechanisms by activating NRF2 nuclear translocation [26]. Since NRF2 is a central transcription factor for antioxidant and anti-ferroptotic genes and GPX4 is an established target gene of NRF2 to prevent lipid peroxidation, we reasoned that the NRF2-GPX4 axis could be a common downstream target of ARID1A and c-MET signaling. To test this hypothesis, we first investigated whether c-MET could regulate the gene expression of GPX4 in CRC cells. Indeed, the c-MET inhibitor PHA significantly downregulated mRNA and protein levels of GPX4 in various CRC lines (Fig. 7A, B). c-MET depletion by siRNA also significantly downregulated GPX4 expression (Fig. 7C). Next, we investigated the combined effects of ARID1A deficiency and c-MET inhibition on GPX4 expression in ARID1A-isogenic CRC cell pairs. PHA treatment downregulated GPX4 levels in *ARID1A*-WT HCT116 cells and this effect was significantly exacerbated in *ARID1A*-KO HCT116 cells (Fig. 7D). Likewise, GPX4 expression was almost completely diminished in ARID1A-mutant RKO cells treated with PHA, compared to PHA-treated ARID1A-overexpressing RKO cells (Fig. 7E). An ectopic overexpression of GPX4 in *ARID1A*-KO HCT116 cells significantly rescued PHA-induced synthetic lethality in ARID1A-deficient cells (Fig. 7F, G). These results suggest that GPX4 is a common transcription target of ARID1A and c-MET signaling, and is a key player mediating the synthetic lethality. As ARID1A is a transcriptional co-regulator that needs to be recruited by a sequence-specific transcription factor on the promoter, we investigated whether ARID1A and NRF2 cooperatively regulate GPX4 expression. Our immunoprecipitation experiment showed that ARID1A interacts with NRF2 (Fig. 7H). Furthermore, NRF2 ChIP experiment showed that NRF2 share the same binding site with ARID1A on the GPX4 promoter (primer #2), suggesting that ARID1A and NRF2 cooperatively regulate the transcription of GPX4 by occupying the same promoter region of GPX4 (Fig. 7I). Interestingly, the binding of NRF2 to GPX4 promoter is significantly reduced in *ARID1A*-KO cells (Fig. 7I). This result raised a possibility that ARID1A is required for NRF2 binding to the GPX4 promoter. NRF2 is known to bind to DNA in a sequence-specific manner, specifically to antioxidant response elements (AREs). In contrast, ARID1A does not exhibit sequence-specific binding, although it does show some specificity for DNA regions such as AT-rich sequences, from which its name is derived. Therefore, it is most likely that NRF2 binds to the GPX4 promoter at the ARE region and recruits ARID1A to the site, rather than ARID1A being required for NRF2 binding to the promoter. The reduction of NRF2 binding to the GPX4 promoter in *ARID1A*-KO cells could be due to the reduced c-MET signaling in these cells. To explore this possibility, we tested the effect of c-MET on NRF2 nuclear localization and its binding to the GPX4 promoter in the presence or absence of ARID1A. Our immunofluorescence experiment showed that NRF2 nuclear localization is partially reduced in *ARID1A*-KO cells compared to wild-type cells (Fig. 7J) and c-MET re-supplementation in *ARID1A*-KO cells significantly rescued the NRF2 nuclear localization phenotype (Fig. 7K). Consequently, c-MET re-supplementation significantly increased NRF2 binding to the GPX4 promoter in *ARID1A*-KO cells (Fig. 7L), suggesting that NRF2 binding to the GPX4 promoter is primarily regulated by c-MET signaling. We further showed that the c-MET inhibitor PHA reduced ARID1A binding to the GPX4 promoter in *ARID1A*-WT cells (Fig. 7M). These results strongly support our notion that c-MET signaling is crucial for NRF2 nuclear translocation and its binding to the GPX4 promoter, and NRF2 in turn recruits ARID1A to the GPX4 promoter to promote GPX4 transcription. In line with the role of c-MET and ARID1A in GPX4 transcription and ferroptosis, we also tested the effect of c-MET re-supplementation on GPX4 inhibitor-induced ferroptotic cell death in *ARID1A*-KO cells. Similar to the data shown earlier (Fig. 3J, K), both Erastin and RSL3 significantly reduced the viability of *ARID1A*-KO cells compared to wild-type cells, while re-supplementation of c-MET significantly rescued RSL3/Erastin-induced cell death (Supplementary Fig. 6), suggesting that c-MET signaling is crucially involved in NRF2-GPX4 axis and ferroptosis regulation in ARID1A-deficient CRC cells. Together, our study shows a functional association among ARID1A, c-MET, and NRF2 in regulating GPX4 transcription and ferroptosis, where ARID1A promotes c-MET expression, which in turn activates NRF2 nuclear translocation. Nuclear NRF2 binds to the GPX4 promoter and recruits ARID1A to the promoter, facilitating SWI/SNF nucleosome remodeler-driven transcription coactivation of GPX4. Consequently, the combination of ARID1A deficiency and c-MET inhibition targets the NRF2-GPX4 axis and induces synthetic lethality by promoting ferroptotic cell death (Fig. 7N).

## Discussion

The SWI/SNF chromatin-remodeling complex orchestrates a wide range of biological pathways by modulating gene expression [1]. ARID1A, an essential component of this complex, possesses a DNA-binding (ARID) domain and plays a pivotal role in directing the complex to gene promoters [40]. Through nucleosome repositioning, ARID1A and the SWI/SNF complex control the transcriptional activity of numerous target genes [41]. Notably, ARID1A can either activate or repress the transcription of target genes. For instance, it can act as a positive regulator of genes such as CDKN1A [42], HDAC6 [28], and HMGCS1 [43], while also functioning as a negative regulator of genes such as GLS1 [29], TERT [44], and AURKA [30]. By regulating the transcription of several key oncogenes or tumor suppressors and multiple other mechanisms, ARID1A is recognized as a bona fide tumor suppressor in various cancer types. Therefore, targeting ARID1A deficiency using a synthetic lethality approach is a promising strategy for cancer treatment. In the present study, we screened a human kinase inhibitor library and identified c-MET as a novel synthetic lethal target for ARID1A in CRC cells. Pharmacological inhibition or siRNA-mediated depletion of c-MET inhibits the growth of ARID1A-deficient CRC cells in vitro and tumor xenografts in vivo. Mechanistically, ARID1A deficiency targets c-MET and GPX4 transcriptions, as well as the expression of iron-exporting protein SLC40A1, thereby increasing the cellular susceptibility to ferroptosis. This could generate cellular dependency on c-MET signaling, where residual c-MET signaling with antioxidant mechanisms could protect ARID1A-deficient cells from ferroptotic cell death. Inhibition of c-MET activity significantly increases intracellular labile iron pool and diminishes GPX4 levels in ARID1A-deficient cells, inducing lipid peroxidation, GSH depletion, and ferroptosis.

Ferroptosis is a type of non-apoptotic, iron-dependent cell death characterized by high iron accumulation and lipid peroxidation during cell death. Ferroptosis inducers diminish cellular glutathione peroxidase activity through multiple pathways, resulting in an impaired cellular antioxidant system and the accumulation of lipid ROS, leading to oxidative cell death [45]. Ferroptosis is closely linked to a variety of pathological conditions, particularly to cancer where the high load of ROS resulting from cancer genetic alterations and abnormal metabolism renders ferroptosis susceptible. Some cancer cells also develop a ferroptosis defense system to survive under metabolic and oxidative stress conditions [46]. Indeed, a number of cancer therapy approaches, such as cancer chemotherapy, radiotherapy, and immunotherapy, kill cancer cells by inducing ferroptotic cell death. Owing to their enormous therapeutic potential, considerable attention has been paid to the development of ferroptosis-targeting drugs for cancer therapy [47]. As a central player in ferroptosis, GPX4 plays a pivotal role in maintaining the cellular redox balance. GPX4 is an antioxidant enzyme that catalyzes the reduction of lipid peroxides at the expense of reduced glutathione (GSH) [48]. As GPX4 protects cells against oxidative stress, its inhibition or inactivation leads to the accumulation of lipid peroxides and ferroptotic cell death [39]. Our study revealed that ARID1A is a transcriptional coactivator of GPX4, and ARID1A-deficient cells have significantly downregulated GPX4 levels. We further showed that ARID1A interacts with the NRF2 transcription factor and occupies the GPX4 promoter to activate its transcription. Therefore, CRC cells lacking ARID1A have increased susceptibility to ferroptosis due to low GPX4 levels. The role of ARID1A in ferroptosis has also been suggested because of its ability to activate the transcription of SLC7A11 [29]. *SLC7A11* encodes a solute carrier family member of the protein that functions as a chloride-dependent cystine-glutamate antiporter. This transporter plays an important role in supplying cysteine, a key source of GSH, to cells. Thus, ARID1A-deficient cells have low GSH levels, which increases their vulnerability to oxidative stress. The combination of low GSH levels and GPX4 activity in ARID1A-deficient cancer cells is thought to significantly increase the susceptibility to ferroptosis. We also found that ARID1A is crucial for NRF2-mediated regulation of target genes beyond SLC7A11 and GPX4, such as HMOX1, FTL, and FTH1. Notably, ARID1A has differential effects on NRF2 target gene transcription. Specifically, ARID1A loss decreased the expression of FTH1, while it increased the levels of FTL and HMOX1 (Supplementary Fig. 7). ARID1A, as a component of SWI/SNF complex, is known to differentially influence target gene transcription in either positive or negative manner. Therefore, the binding of ARID1A to NRF2 appears to have differential effects on NRF2 target genes depending on the nucleosome positions.

c-MET, a receptor tyrosine kinase that induces a wide range of biological pathways involved in cancer growth, is overexpressed in many types of cancers. c-MET activation is involved in drug resistance by protecting cells from the antitumor effects of chemotherapy and targeted therapy drugs. A recent study demonstrated that c-MET activation protected renal cancer cells from sorafenib-induced oxidative stress and cytotoxicity through activation of the NRF2-HO-1 antioxidant pathway [26]. Furthermore, liver-specific knockout of c-MET in mice resulted in overproduction of ROS, increased lipid peroxidation, and reduced GSH content. Treatment of primary hepatocytes isolated from c-MET-KO mice with N-acetylcysteine, an antioxidant GSH precursor, protects cells from the cytotoxic effects of anticancer drugs, while a GSH-depleting agent promotes cell death [49], suggesting a pivotal role of c-MET in cellular defense against oxidative stress. In the present study, we showed that ARID1A loss reduces c-MET expression and GPX4 levels, thus making cells hyper-vulnerable to agents that induce oxidative stress and ferroptosis. Residual c-MET activity with the minimal GPX4 level in ARID1A-deficient cells is thought to play a critical role in cell survival since the inhibition of c-MET activity or siRNA depletion completely abolished GPX4 expression and induced ferroptosis. Ectopic overexpression of GPX4 significantly rescued the c-MET inhibitor-induced cell death, supporting our model. Our further mechanistic study revealed a tripartite functional association among ARID1A, c-MET, and NRF2, where ARID1A and c-MET signaling cooperatively regulate NRF2 transcription factor for GPX4 transcription and ferroptosis in CRC cells.

In conclusion, this study identified a synthetic lethal association between ARID1A and c-MET, where ARID1A-deficient CRC cells are highly dependent on c-MET activity to survive ferroptotic cell death. Our findings establish c-MET kinase and ferroptosis pathways as druggable targets capable of inducing synthetic lethality in ARID1A-deficient CRC. Notably, FDA-approved c-MET inhibitors, such as crizotinib and cabozantinib, have demonstrated enhanced cytotoxicity in ARID1A-deficient cancers, facilitating the translation of these approved drugs for repurposing in ARID1A-targeted precision cancer medicine.

## Supplementary information

Supplementary data

Original Data Files

Supplementary Table 1

## Data Availability

All supporting data are available within the article and supplementary files, or corresponding authors upon reasonable request.
