# Supplementary material for: ARID1A loss enhances sensitivity to c-MET inhibition by dual targeting of GPX4 and iron homeostasis, inducing ferroptosis
Source: Cell Death Differ. 2025 May 14;32(11):2009–21. doi: 10.1038/s41418-025-01510-x (PMC12572266; doi:10.1038/s41418-025-01510-x)
Supplement: Supplementary file 1 — Supplementary data [file 41418_2025_1510_MOESM1_ESM.docx]

**Supplementary Fig. 1**

| 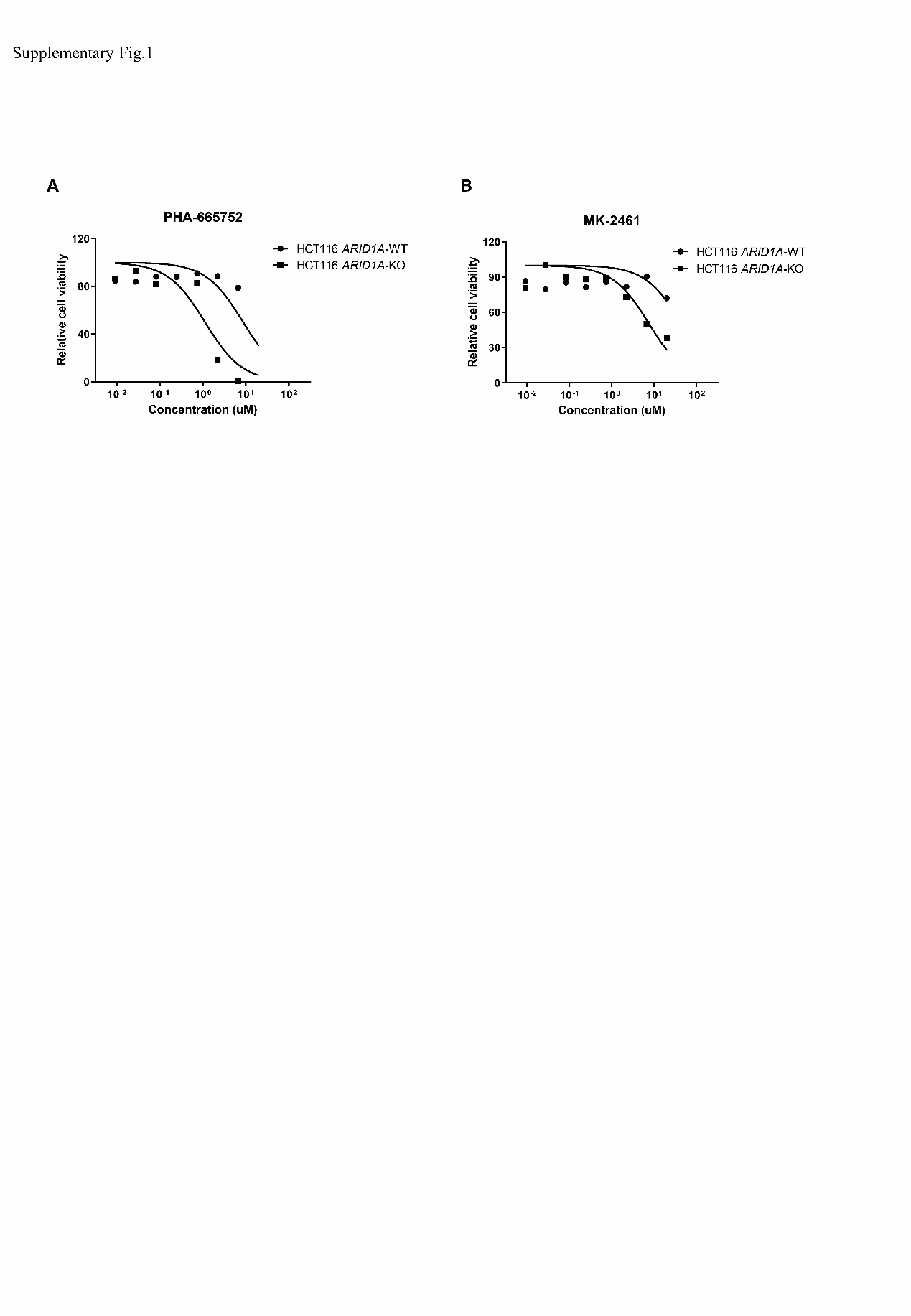 |
| --- |
| **Supplementary Fig. 1** Dose-response curve of PHA-665752 (A) and MK-2461 (B). |

**Supplementary Fig. 2**

| 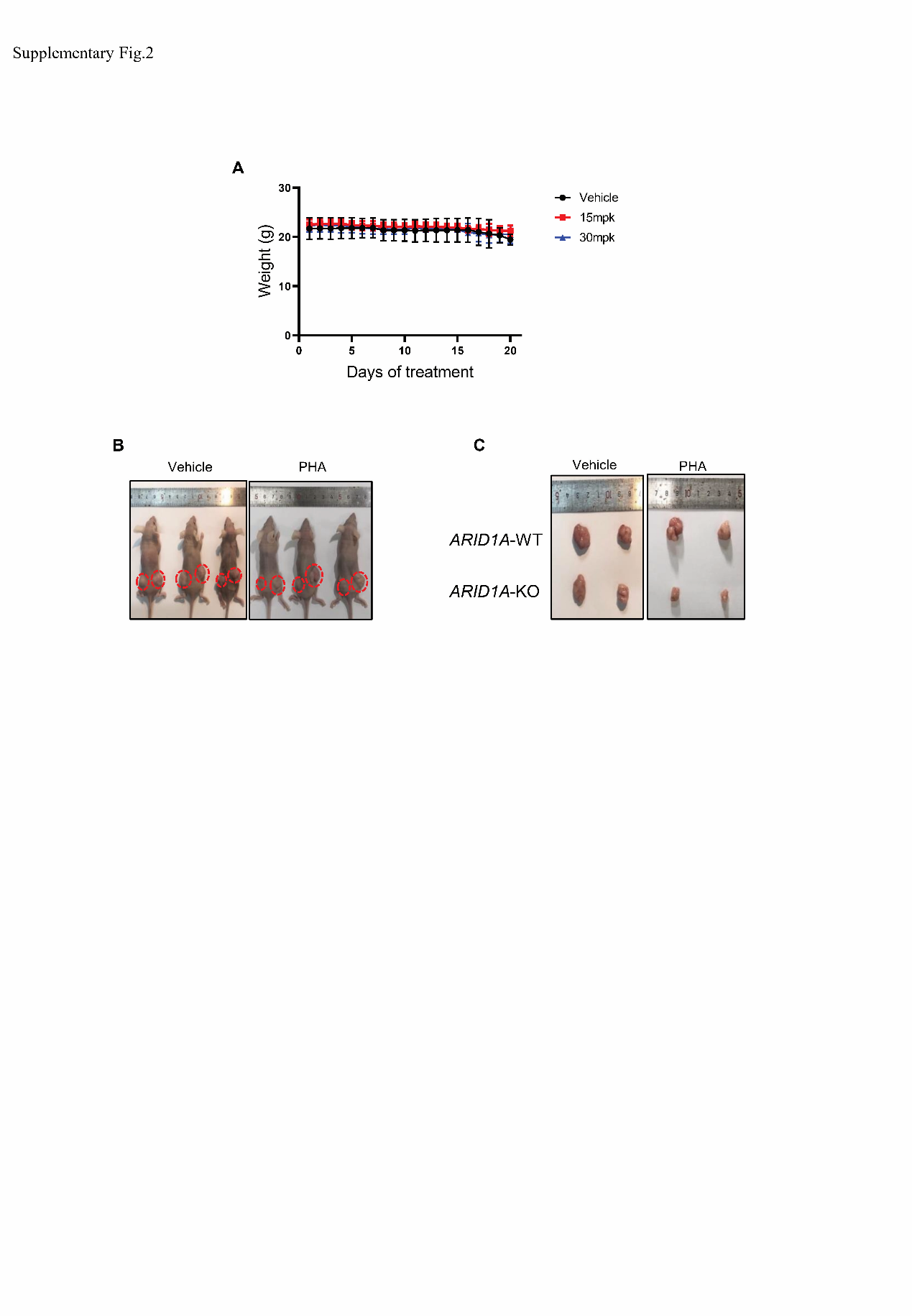 |
| --- |
| **Supplementary Fig. 2** **(A)** Body weight of mice treated with PHA for 20 days. **(B)** Representative images of vehicle-treated and PHA-treated nude mice bearing HCT116 WT and *ARID1A*-KO xenografts. **(C)** Representative images of tumor size with or without PHA treatment. |

**Supplementary Fig. 3**

| 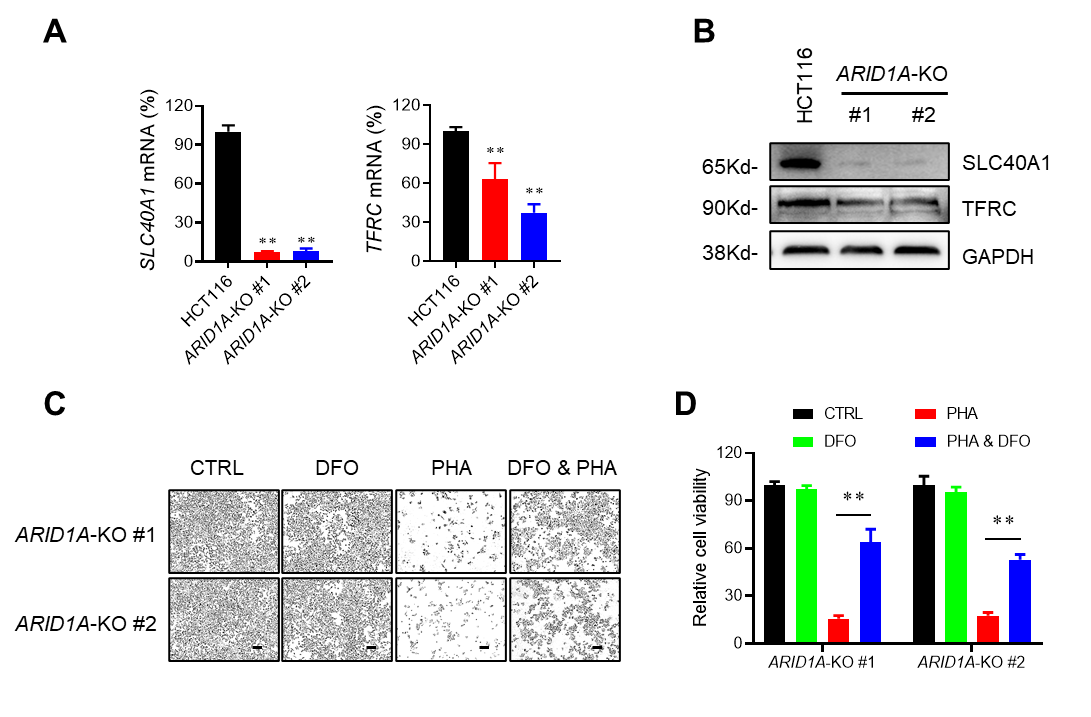 |
| --- |
| **Supplementary Fig. 3 ARID1A regulates iron transport genes expression and DFO reversed PHA-induced cell death in *ARID1A*-KO cells.** **(A)** RT-qPCR analysis of *SLC40A1*, *STEAP3*, and *TFRC* mRNA level in HCT116 and *ARID1A*-KO clones. ANOVA P < 0.01. **(B)** Immunoblot analysis showing ARID1A loss downregulates FPN, STEAP3 and TFRC level in HCT116 cells. **(C-D)** DFO reversed PHA-induced cell death in ARID1A-KO cells. HCT116 isogenic cells were treated with or without PHA and DFO. The cell viability was determined by Image J software. ANOVA P value of <0.01. |

**Supplementary Fig. 4**

| 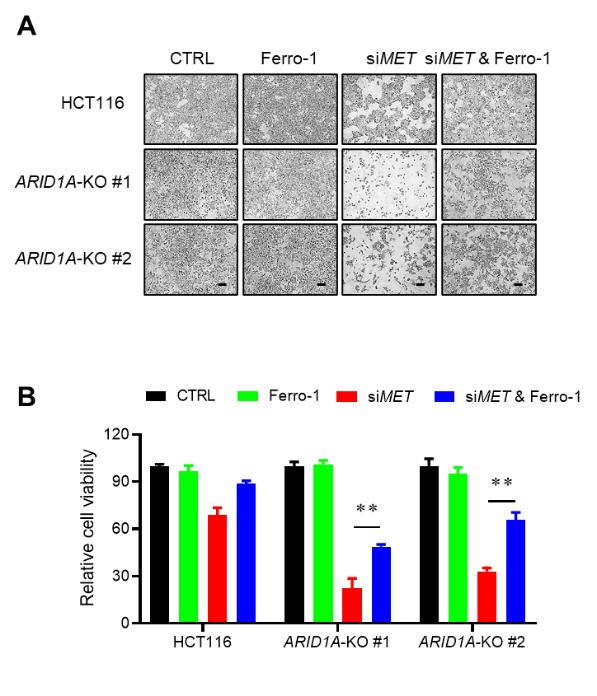 |
| --- |
| **Supplementary Figure 4. Ferrostatin-1 can rescue c-MET silence induced cell death.** HCT116 and *ARID1A*-KO cells were transfected with or without siMET and then treatment with or without Ferrostatin-1. The cell viability was determined by Image J software. |

**Supplementary Fig. 5**

| 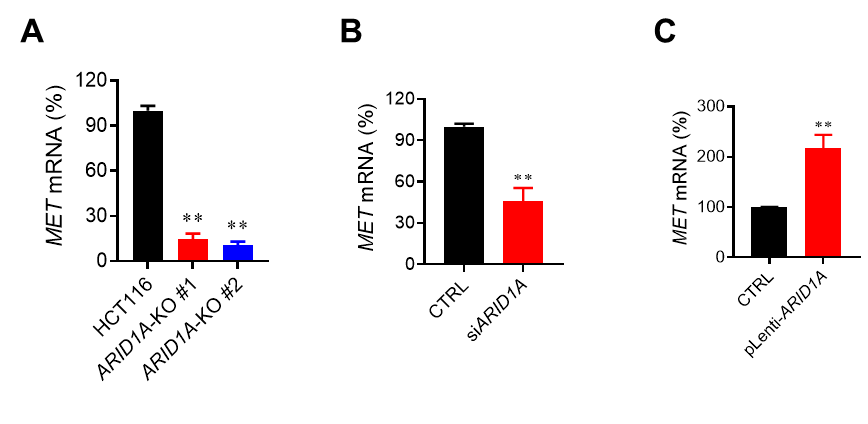 |
| --- |
| **Supplementary Fig. 5 ARID1A regulates *MET* mRNA level.** (a) RT-qPCR analysis of *MET* level in HCT116 and *ARID1A*-KO clones. ANOVA P < 0.01. (b) RT-qPCR analysis of *MET* mRNA level with siARID1A in HCT116 cells. ANOVA P < 0.01. (C) RT-qPCR analysis of *MET* mRNA level with pLenti-ARID1A overexpresion in HCT116 cells. ANOVA P < 0.01. |

**Supplementary Fig. 6**

| 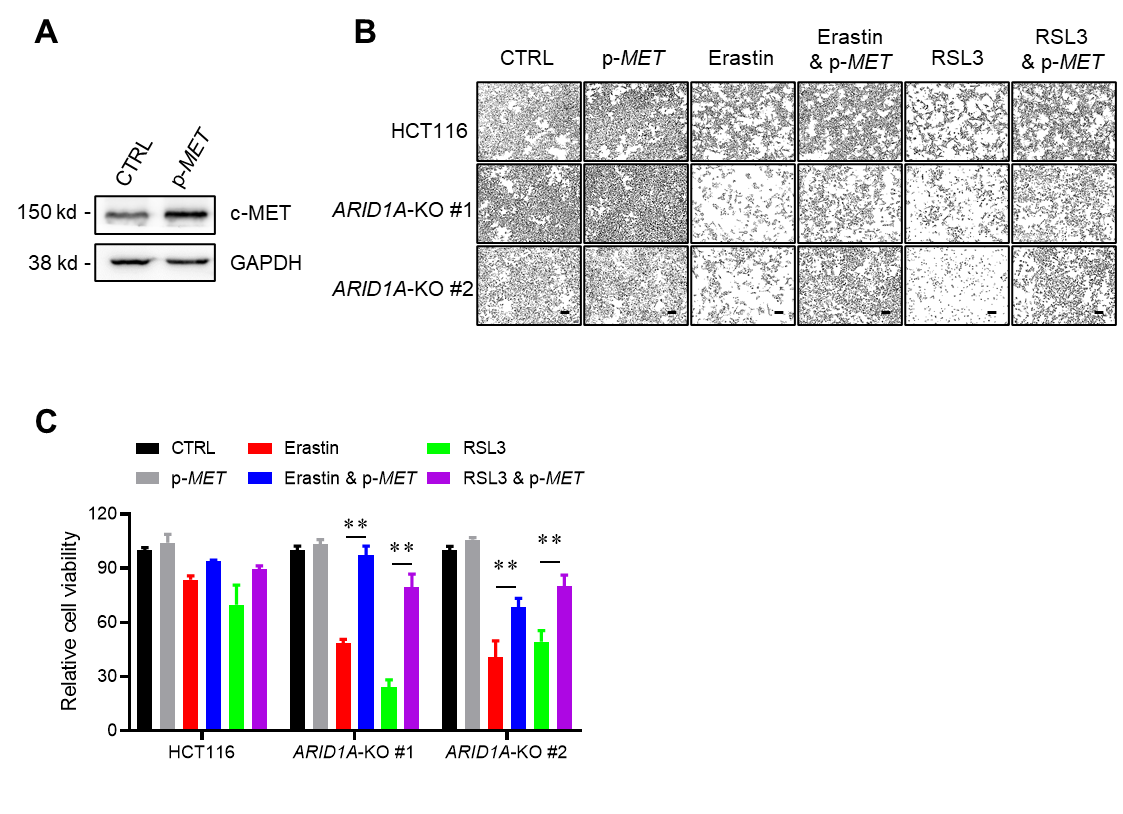 |
| --- |
| **Supplementary Fig. 6 Overexpression of c-MET reversed RSL3/Erastin-induced ferroptosis.** HCT116 isogenic cells were treated with or without RSL/Erastin and c-MET expresion plasmid. The cell viability was determined by Image J software. ANOVA P value of <0.01. |

**Supplementary Fig. 7**

| 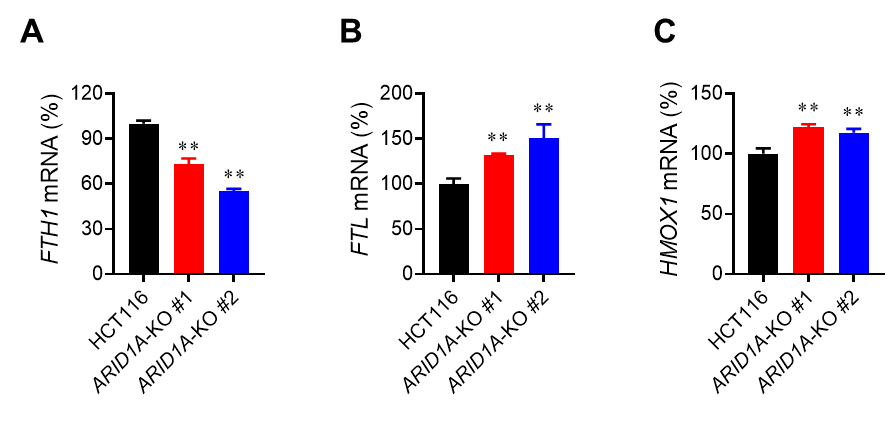 |
| --- |
| **Supplementary Fig. 7 ARID1A regulates NRF2 targets.** RT-qPCR analysis of *FTH1*, *FTL*, and *HMOX1* mRNA level in HCT116 and *ARID1A*-KO clones. ANOVA P < 0.01. |
