# Supplementary material for: ARID1A loss enhances sensitivity to c-MET inhibition by dual targeting of GPX4 and iron homeostasis, inducing ferroptosis
Source: Cell Death Differ. 2025 May 14;32(11):2009–21. doi: 10.1038/s41418-025-01510-x (PMC12572266; doi:10.1038/s41418-025-01510-x)
Supplement: Supplementary file 2 — Original Data Files [file 41418_2025_1510_MOESM2_ESM.docx]

**Supplementary Fig. 8**

| 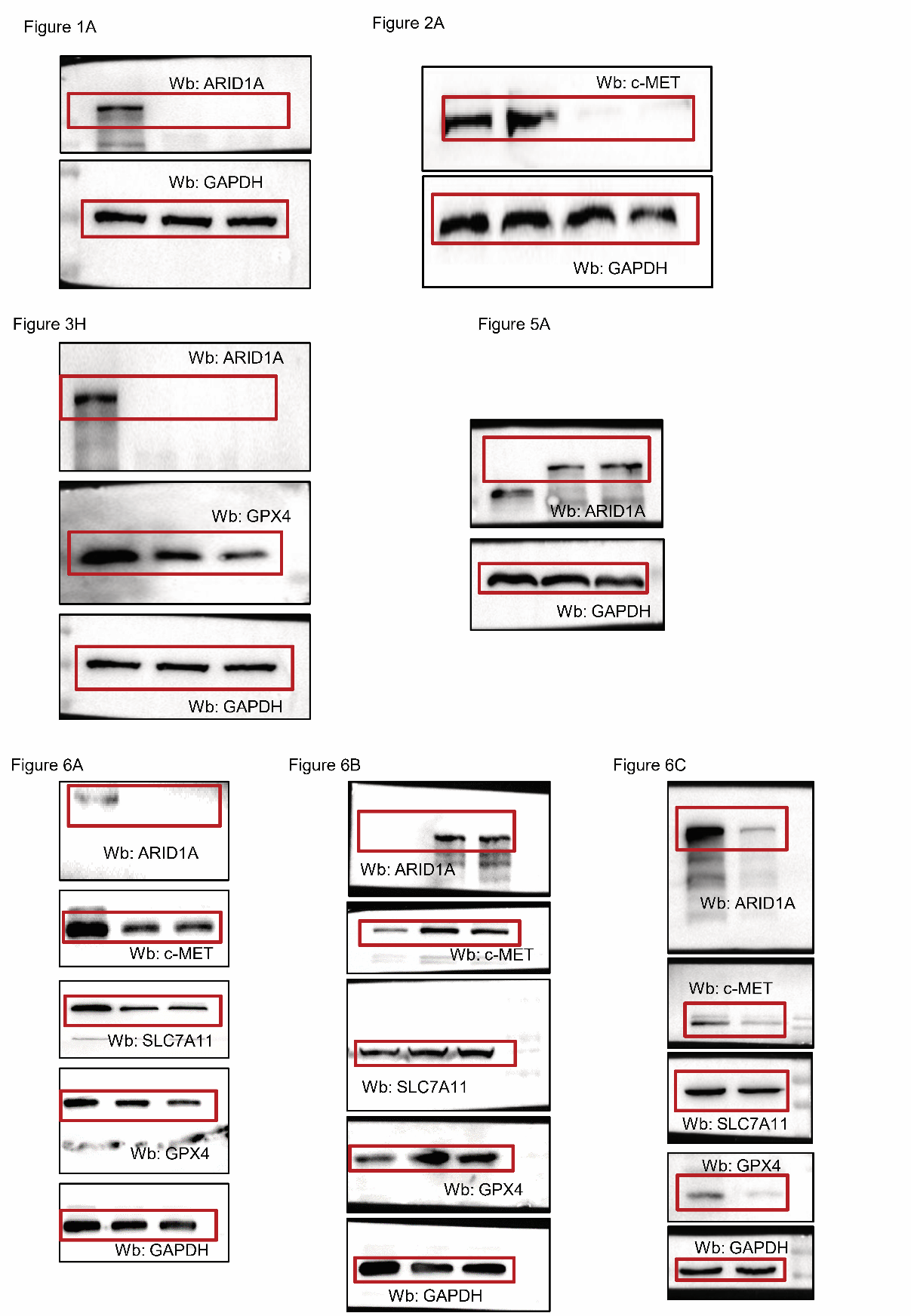 |
| --- |
| **Supplementary Fig. 8 Original Western Blot shown in Figures 1-6.** Each figure corresponds to the Western Blots in the indicated Figure number. |

**Supplementary Fig. 9**

| 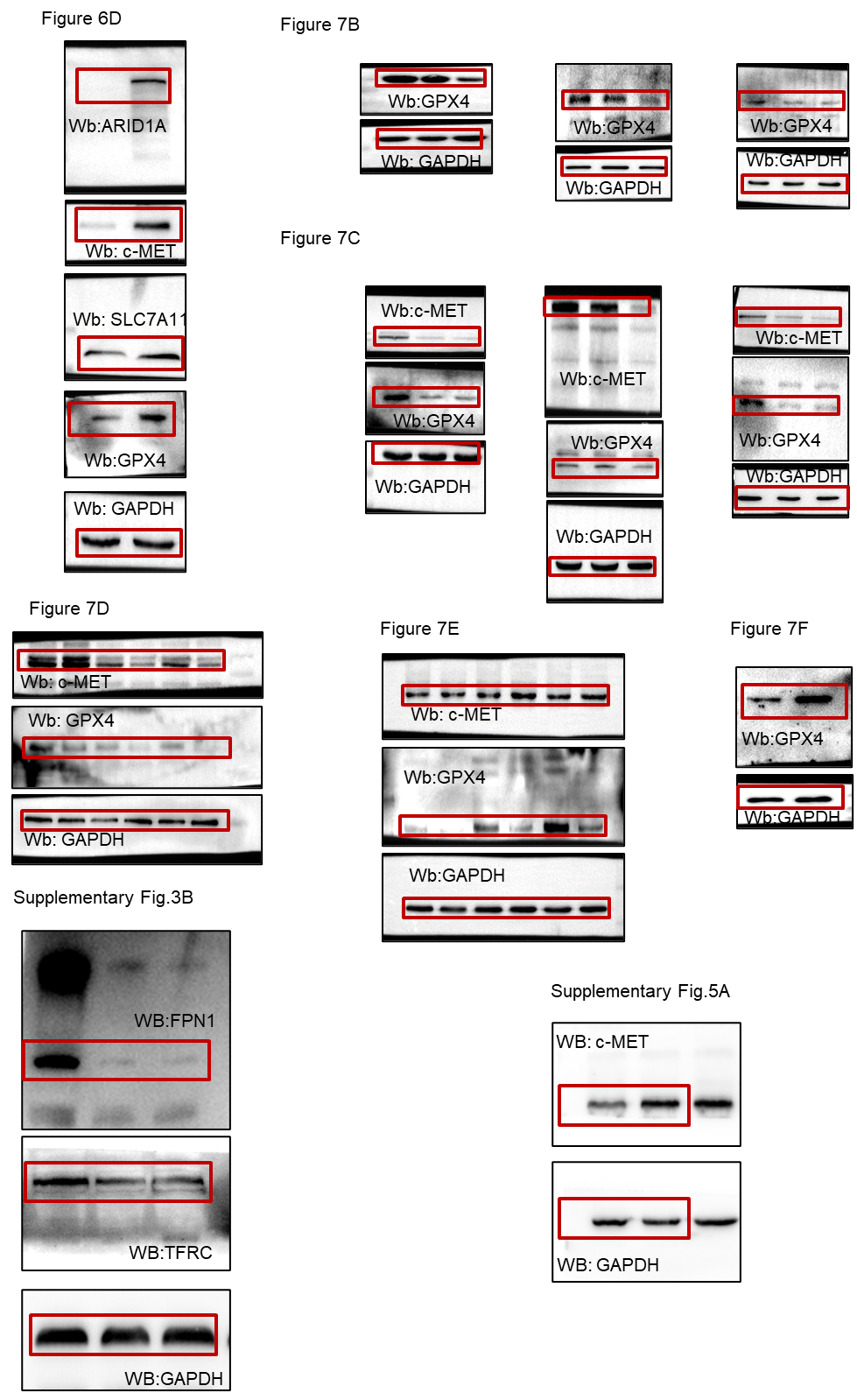 |
| --- |
| **Supplementary Fig. 9 Original Western Blot shown in Figures 6-7, and supplementary Figures.** Each figure corresponds to the Western Blots in the indicated Figure number. |
