# Supplementary material for: ARID1A loss enhances sensitivity to c-MET inhibition by dual targeting of GPX4 and iron homeostasis, inducing ferroptosis
Source: Cell Death Differ. 2025 May 14;32(11):2009–21. doi: 10.1038/s41418-025-01510-x (PMC12572266; doi:10.1038/s41418-025-01510-x)
Supplement: Supplementary file 3 — Supplementary Table 1 [file 41418_2025_1510_MOESM3_ESM.docx]

Supplementary Table 1. Primer sequences used for real-time quantitative reverse transcriptase polymerase chain reaction (qRT-PCR)

| *Gene* | *5’ to 3’* | *Primers* |
| --- | --- | --- |
| *GPX4* | Sense | GAGGCAAGACCGAAGTAAACTAC |
|  | Anti-Sense | CCGAACTG GTTACACGGGAA |
| *SLC40a1* | Sense | CTACTTGGGGAGATCGGATGT |
|  | Anti-Sense | CTGGGCCACTTTAAGTCTAGC |
| *TFRC* | Sense | ACCATTGTCATATACCCGGTTCA |
|  | Anti-Sense | CAATAGCCCAAGTAGCCAATCAT |
| *FTL* | Sense | CAGCCTGGTCAATTTGTACCT |
|  | Anti-Sense | GCCAATTCGCGGAAGAAGTG |
| *FTH1* | Sense | CCCCCATTTGTGTGACTTCAT |
|  | Anti-Sense | GCCCGAGGCTTAGCTTTCATT |
| *MET* | Sense | GAGAAGCCCAAGCCCATCC |
|  | Anti-Sense | GCCCAGGGCTCAGAGCTT |
| *HMOX1* | Sense | GGCCTCCCTGTACCACATCT |
|  | Anti-Sense | CTGCATGGCTGGTGTGTAGG |
